# Extraction, Structure and Immunoregulatory Activity of Low Molecular Weight Polysaccharide from *Dendrobium officinale*

**DOI:** 10.3390/polym14142899

**Published:** 2022-07-16

**Authors:** Su-Jun Sun, Peng Deng, Chun-E Peng, Hai-Yu Ji, Long-Fei Mao, Li-Zeng Peng

**Affiliations:** 1 Key Laboratory of Agro-Products Processing Technology of Shandong Province, Key Laboratory of Novel Food Resources Processing Ministry of Agriculture, Institute of Agro-Food Science and Technology, Shandong Academy of Agricultural Sciences, Jinan 250100, China; sunsujun4307@saas.ac.cn (S.-J.S.); dengpeng2017@saas.ac.cn (P.D.); pengchune1203@saas.ac.cn (C.-E.P.); longfeimao1988@saas.ac.cn (L.-F.M.); 2 College of Life Sciences, Yantai University, Yantai 264005, China; jihaiyu@ytu.edu.cn

**Keywords:** *D. officinale*, low-molecular weight polysaccharide, structural characteristics, immunoregulatory activity

## Abstract

The ethanol precipitation method has been widely-used for *Dendrobium officinale* polysaccharides preparation. However, the alcohol-soluble fractions have always been ignored, which causes significant wastes of resources and energies. In this study, the extraction, physicochemical properties, and immune regulation activity of an edible *D. officinale* polysaccharide (DOPs) isolated from the supernatant after 75% ethanol precipitation were systematically investigated. The structural characteristics determination results showed that DOPs was mainly composed of glucose and mannose at a molar ratio of 1.00:5.78 with an average molecular weight of 4.56 × 10^3^ Da, which was made up of α-(1,3)-Glc*p* as the main skeleton, and the α-(1,4)-Glc*p* and β-(1,4)-Man*p* as the branches. Subsequently, the cyclophosphamide (CTX)-induced immunosuppressive mice model was established, and the results demonstrated that DOPs could dose-dependently protect the immune organs against CTX damage, improve the immune cells activities, and promote the immune-related cytokines (IL-2, IFN-γ and TNF-α) secretions. Furthermore, DOPs treatment also effectively enhanced the antioxidant enzymes levels (SOD, GSH-Px) in sera and livers, therefore weakening the oxidative damage of CTX-treated mice. Considering these above data, DOPs presented great potential to be explored as a natural antioxidant and supplement for functional foods.

## 1. Introduction

*Dendrobium officinale*, the rhizome of *D. officinale* Kimura et Migo in the orchid family, is a kind of well-known edible plants with superior biological functions including anticancer, immunoenhancing, antioxidant, hepatoprotective effects and so forth [1,2]. At present, *D. officinale* is generally added to beverages, tea, and soup as healthy complementary food [3]. The main bioactive substances of *D. officinale* contain polysaccharides, dendrobine, phenanthrenes, amino acids, and multiple trace mineral elements [4]. Among these medicinal components, polysaccharides are regarded as the leading active substances of *D. officinale*, which possess immunomodulatory, anti-tumor, anti-inflammation, hypoglycemic effects, and so forth [5,6,7,8]. The contents of biological polysaccharides play a crucial role in the quality of *D. officinale*. Given this, the extraction and isolation of *D. officinale* polysaccharides have drawn more and more attentions all over the world.

Increasing evidences demonstrated that the biological activities of natural polysaccharides are closely correlated with their molecular weight, functional groups, sugar composition, and other physicochemical and structural properties [9,10]. And different extraction methods and processing procedures have an important influence on these above properties of polysaccharides [11]. Recently, the extraction technologies for the *D. officinale* polysaccharides are mainly hot water-extraction and alcohol-precipitation, or assisted with ultrasonic, microwave, enzyme, and other modern extraction techniques [12]. In this process of extraction, the polysaccharide precipitation with higher molecular weight was obtained as main biological ingredients. However, the fractions dissolved in ethanol were discarded, thereby resulting in the utilization reduction and the waste of natural sources. Studies have indicated that the polysaccharides in the ethanol supernatant possessed the relatively lower molecular weight and superior biological activities [13,14]. To make the utmost of *D. officinale*, it is urgently necessary for us to isolate the biological polysaccharides from the supernatant after ethanol precipitation.

Immune system is very important for human body to perform immune response and immune function, which is mainly regulated by immune organs, cells and active molecules [15]. Immune disorders can be caused by many factors including malnutrition, unhealthy emotions, great pressure, chemotherapy, etc. [16]. Increasing evidence has indicated that natural plant polysaccharides possessed superior immunomodulatory effects. Additionally, numerous plant polysaccharides possess obvious oxidation resistances, which can protect the body against free radical damage, and decrease the disease risk [17,18]. The abundant antioxidant capacity of polysaccharides had a clear impact on their effective application as supplements in pharmaceuticals and health foods fields [19]. Research showed that the biological activities of *D. officinale* polysaccharides were closely associated with their sugar content and relative molecular weight [20]. However, rare researches focused on the *D. officinale* polysaccharides isolated from the supernatant after 75% ethanol precipitation and their immunoregulatory and antioxidant potentials in vivo.

In the present study, a novel polysaccharide (DOPs) from *D. officinale* was obtained from 75% ethanol-dissolved fractions after hot-water extraction (80 °C) and 75% ethanol treatment. The structural properties of DOPs were characterized, and the immunoregulatory and antioxidant effects on cyclophosphamide induced immunosuppressed mice were further investigated. These findings might offer novel insights into the effective use of *D. officinale* and the practical applications of DOPs in functional foods and pharmaceuticals fields.

## 2. Materials and Methods

### 2.1. Materials and Regents

The dried powder of D. officinale were purchased from Huoshan (sun clara). T-series dextrans and monosaccharide standards were provided by Sigma-Aldrich Co. (St. Louis, MO, USA). 3-(4,5-dimethyl-2-thiazolyl)-2,5-diphenyl-2-H-tetrazolium bromide (MTT), dimethyl sulfoxide (DMSO), ConA, LPS and neutral red were obtained from Solarbio biological technology Co., Ltd. (Beijing, China). Mouse IL-2, TNF-α and IFN-γ ELISA Kits were provided from ZCIBIO Technology Co., Ltd. (Shanghai, China). All of other chemicals were of analytical grade. Superoxide Dismutase (SOD), Glutathione Peroxidase (GSH-Px) and Malondialdehyde (MDA) assay kits were purchased from Nanjing Jiancheng Bioengineering Institute (Nanjing, China). All of other chemicals were of analytical grade.

### 2.2. Preparation of D. officinale Polysaccharide

The powder of *D. officinale* was soaked in hot distilled water (1:10, *w*/*v*) at 80 °C for 3.0 h, twice. The supernatant was collected by centrifugation (3500 rpm, 10 min), and concentrated by a rotary evaporation (60 °C, vacuum), and precipitated by addition of three times volume of absolute ethanol (4 °C, 12 h). The precipitation was removed by centrifuging at 3500 rpm for 10 min, and the fractions were collected from the alcohol-dissolved supernatant. After the ethanol was discarded by rotary evaporation, the deproteinization was further carried out using Sevag method as described previously [21]. After the Sevag regents were removed, the water-soluble fraction was dialyzed (MWCO, 1000 Da) against distilled water for three days, and freeze-dried to produce the crude *D. officinale* polysaccharide (cDOP).

The cDOP aqueous solution (10 mg/mL) was separated and purified by loading on a sephadex-G25 column, and the distilled water was used as the elution solution. The total sugar and reducing sugar contents of each tube were monitored by phenol-sulfuric acid method [22] and DNS method [23], respectively. The major polysaccharide fractions containing no reducing sugar were collected and freeze-dried to obtain the purified *D. officinale* polysaccharide (named as DOPs) for subsequent structural and biological activities analysis.

### 2.3. Chemical Components Analysis of DOPs

The total sugar, protein and uronic acid contents of DOPs were determined based on corresponding methods of phenol-sulfuric acid [22], coomassie brilliant blue [24], m-hydroxyl diphenyl [25], respectively. Moreover, UV-2500PC UV-Vis spectrophotometer (Shimadzu, Japan) was applied to demonstrate the presence or absence of protein and nucleic acid in DOPs by scanning from 200 nm to 800 nm.

### 2.4. Determination of Average Molecular Weight of DOPs

High performance gel permeation chromatography (HPGPC, Agilent, CA, USA) was used to measure the average molecular weight of DOPs according to previous method [26]. 20 μL of DOPs aqueous solution (2 mg/mL) was separated by TSK-gel G4000PWxL column (7.8 mm × 300 mm) with the column temperature of 30 °C. The ultrapure water was used as eluent with the flow rate of 0.8 mL/min, and the refractive index detector (RID) as the detector at 35 °C. The average molecular weight of DOP was calculated based on the calibration curve, which was established according to T-series dextran with known molecular weights.

### 2.5. Determination of Monosaccharide Composition of DOPs

Gas chromatography (GCMS7890B-7000C, Agilent, CA, USA) was applied to investigate the monosaccharide composition and corresponding molar ratio of DOPs. Initially, 5 mg of dried DOPs sample was distilled in 1 mL of trifluoroacetic acid (2 mol/L), hydrolyzed at 110 °C for 4 h, and acetylated according to previous literatures [27]. The derivative products were detected by using GC. The monosaccharide components were identified by comparison with the derivatives of six monosaccharide standards (D-glucose, D-mannose, D-arabinose, D-xylose, D-galactose and L-rhamnose).

### 2.6. FT-IR Analysis of DOPs

Fourier transform infrared spectroscopy (FT-IR, Bruker, Karlsruhe, Germany) was applied to identify the functional groups of DOPs. In brief, approximately 1.0 mg of dried DOPs powder and 150 mg of KBr powder were mixed and milled adequately. Subsequently, the mixture was crushed into a slice and determined on an VECTOR-22 infrared spectrometer in the range from 4000 to 400 cm^−1^.

### 2.7. NMR Analysis of DOPs

The ^1^H, COSY, HSQC and HMBC NMR spectra of DOPs were determined by Advance DPX-400 NMR spectrometer (400 MHz) (Bruker, Karlsruhe, Germany) to further analyze the structural properties employing 99.9% D_2_O as solvent, subsequently the data was analyzed through TopSpin 4.1.0 software.

### 2.8. Animal Grouping and Experimental Design

Sixty female Kunming mice (six to eight weeks old, body weight 20−25 g) were purchased from SPF (Beijing) Biotechnology Co., Ltd. (Beijing, China) with a production license number of SYXK(Jing) 2019–0010, and raised in the experimental animal room with relative humidity of 45~55%), and a controllable temperature of 20~25°C. After adapting for seven days, these mice were randomly separated into blank control group, model group, positive control group (cyclophosphamide, CTX, 30 mg/kg), low-dose DOPs group (50 mg/kg DOPs), middle-dose DOPs group (100 mg/kg DOPs) and high-dose DOPs group (200 mg/kg DOPs) with 10 mice in each group. The mice were orally treated with 0.2 mL of 0.9% normal saline in blank control group, model group, positive control group; the mice in low-, middle- and high-dose DOPs groups were administrated with 50, 100 and 200 mg/kg/d DOPs, respectively, via gavage. After seven days of gavage, all of the experimental groups except the blank control group were peritoneal injection with 30 mg/kg/d CTX for 15 days; simultaneously, the intragastric administration was performed continuously for the same time. During the experiment, all of the mice were allowed to take water and food freely.

### 2.9. Assays of Body Weights and Organs Indices

After the last administration, all of the animals were fasted for 12 h. All mice were weighted and recorded. Blood samples were obtained from mice eyeball, stood for at least 4 h, followed by centrifugation (4000 rpm, 10 min) at 4 °C to collect the supernatant serum. The mice were sacrificed, livers, thymuses and spleens were dissected, washed with pre-cold 0.9% normal saline, sopped up and weighted immediately. The organ indices were calculated as the ratios of organ weights (mg) to body weights (g).

### 2.10. Assay of Splenic Lymphocyte Proliferation

The proliferative activities of splenic T lymphocytes and B lymphocytes were firstly evaluated by using ConA and LPS as the stimuluses. The obtained spleen tissues were milled slightly with PBS, and then filtrated by cell filtrator to get the single-cell suspension under aseptic condition. After washed with PBS three times, the splenocytes were collected by centrifugation at 1000 rpm for 5 min, and adjusted the cell density into 1 × 10^7^ cells/mL with RPMI-1640 medium. Results of trypan-blue stain suggested that 95% of the cells were viable. 100 µL/well freshly-prepared cell suspension was added into 96-well plates, and 100 µL/well Con A (final concentration of 5 μg/mL) and 100 µL/well LPS (final concentration of 10 μg/mL) were seeded into corresponding holes, respectively, for 48-h cocultivation under a condition of 37 °C, 5% CO_2_. Equivoluminal RPMI-1640 medium replaced LPS or ConA as the control. After that, 10 µL MTT solution (5 mg/mL in PBS) was added into each well for another 4 h, followed by the removal of the supernatant and the addition of 150 µL/well DMSO. The optical density (OD) was determined at 570 nm, and the stimulation index (SI) was calculated as following:

SI = OD_1_/OD_2_ (1)

where OD_1_ and OD_2_ showed the absorbance of LPS/ConA groups and control group (RPMI-1640 medium), respectively.

### 2.11. Assay of Natural Killer (NK) Cells Activity

The splenocytes were obtained as the effector cells according to the above method, and the murine liver cancer H22 cells were used as target cells for the evaluation of cytotoxicity of splenic NK cells according to Wang’s et al. method [28] with some slight modifications. In brief, 100 µL splenocytes (5 × 10^6^ cells/mL) were added into each well of 96-well plates, and co-cultured with H22 cells (ratio of 20:1) for 48 h under a condition of 37 °C, 5% CO_2_. The splenocytes or H22 cells cultured with RPMI 1640 medium only were used as the control. An MTT assay was used to evaluate the cell viabilities, and the OD values were determined at 570 nm. The cytotoxicity of splenic NK cells was calculated according to the Formula:

Cytotoxicity (%) = (OD_E_ + OD_T_ − OD_C_)/OD_E_ × 100 (2)

where OD_E_, OD_T_, and OD_C_ showed the absorbance of H22 cells, NK cells and co-cultured cells, respectively.

### 2.12. Assay of Macrophages Phagocytosis

The macrophages phagocytosis was investigated by using neutral red as reported previously by Xiong’S et al. [29] with some modifications. After the mice were euthanized, 5 mL PBS buffer was injected into the abdominal cavity, followed by gentle kneading for 5 min. The Abdominal cavity fluid was collected and centrifuged at 1000 rpm for 5 min to obtain the peritoneal macrophages. The macrophages were washed with PBS three times, re-suspended with RPMI-1640 medium, and adjusted into 1 × 10^5^ cells/mL. 100 μL of cell suspension were seeded to each well of 96-well plates, and cultured for 24 h in a cell incubator with 5% CO_2_ at 37 °C. After 24 h, the supernatant was abandoned, and 100 μL neutral red solution (0.075%, m/v) was added into each well for another 2-h incubation. Subsequently, the cells were washed with PBS three times, and then lysed by an addition of 100 µL/well of cell lysis buffer. Following the plates were vortexed thoroughly for 10 min, the absorbance was detected at 540 nm.

### 2.13. Assay of Cytokines in Sera

The levels of immune cytokines (IL-2, IFN-γ, TNF-α) in serum were evaluated by using commercial enzyme-linked immunosorbent assay (ELISA) kits (Jiancheng Bioengineering Institute, Nanjing, China) based on the instructions. The amounts of the cytokines were obtained according to the corresponding standard curves.

### 2.14. Assays of Antioxidant Activities

The livers were homogenized rapidly with pre-cold 0.9% normal saline to make into homogenate (0.1 g tissues/mL), centrifuged (4000 rpm, 10 min) at 4 °C to collect the supernatant. All freshly-prepared samples were stored at −80 °C for following researches.

The activities of superoxide dismutase (SOD), glutathione peroxidase (GSH-Px) and malondialdehyde (MDA) contents in sera and livers were detected using corresponding detection kits based on the manufacturer’s operating instructions.

### 2.15. Statistical Analysis

All values in this study were expressed as the mean  ±  standard deviation (S.D.). The significance of between-group variance was determined by student’s *t*-test and one-way analysis of variance (ANOVA), and the value of *p*  <  0.05 was regarded to be significant.

## 3. Results and Discussion

### 3.1. Purification, Chemical Constitutions, and Average Molecular Weight of DOPs

**Figure 1:**
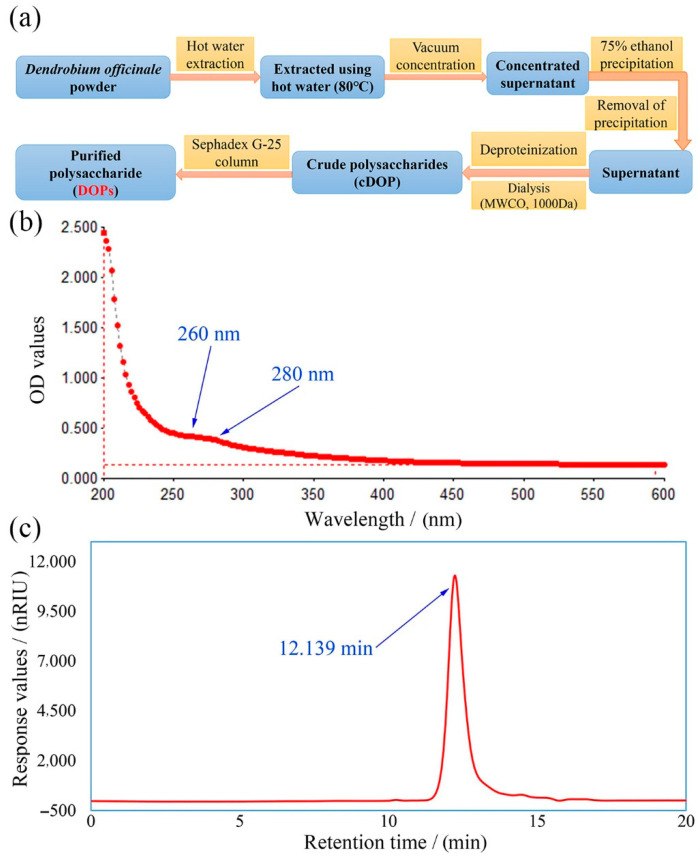
The flow diagram of extraction and purification process for DOPs (**a**); UV-vis spectrum (**b**) and HPGPC profile (**c**) in DOPs.

As shown in Figure 1a, the crude *D. officinale* polysaccharides (cDOP) were prepared from the ethanol-soluble supernatant after hot-water (80 °C) extraction and three volumes of ethanol precipitation. After deproteinization and dialysis, the cDOP were further isolated and purified by using Sephadex G-25 column to obtain the main purified fraction (DOPs), with a yield of 2.76 ± 0.13%, which was calculated based on the weight of dried *D. officinale* powder.

According to the results of chemical components analysis, the contents of total sugar, protein and uronic acid in DOPs were calculated as 93.72 ± 2.06%, 1.34 ± 0.08% and 0.57 ± 0.02%, respectively, indicating that DOPs was a neutral polysaccharide. Moreover, UV-Vis spectrum (Figure 1b) showed no evident absorbance peaks at 260 nm and 280 nm, indicating the absence of nucleic acid and proteins in DOPs, which was in accordance with the above chemical constitutions analysis.

As seen from Figure 1c, a single symmetrical peak was observed in HPGPC profiles, verifying that DOPs was a uniform polysaccharide. According to the regression equation established by the retention time as x-coordinate and the molecular weight on a log scale as y-coordinate based on serial Dextran standards, the average molecular weight of DOPs was evaluated to be 4.56 × 10^3^ Da. As reported, the molecular weights of the polysaccharides from *D. officinale* mainly distributed in the range from 30,000 to 1,415,000 Da [12]. In present study, the relatively low molecular weight might be due to the different raw material resources, as well as extraction method and isolation process.

### 3.2. Monosaccharide Composition and FTIR Analysis

**Figure 2:**
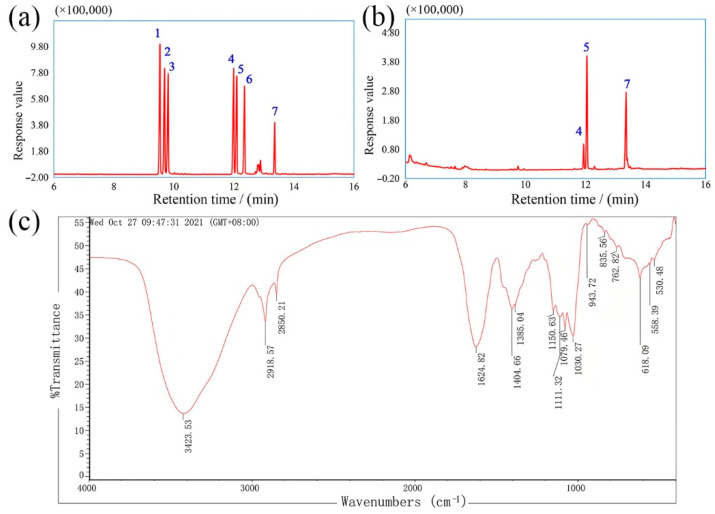
GC spectra of six monosaccharide standards (**a**) and DOPs (**b**) (Peaks identity: 1- L-rhamnose; 2- D-arabinose; 3- D-xylose; 4- D-mannose; 5- D-glucose; 6- D-galactose; 7- myo-inositol hexaacetate); FT-IR spectrum (**c**) of DOPs.

The sugar composition and the molar ratio of DOPs were identified and analyzed by GC-MS. As shown in Figure 2a,b, DOPs was mainly made up of glucose and mannose, by comparison with the retention times of six monosaccharide standards. The monosaccharide constitutes were consistent with the reported polysaccharides from *D. officinale* [30,31]. According to the peak areas, the molar ratio of glucose and mannose was calculated to be 1.00:5.78. The results showed that different extraction and purification processes had less influence on the monosaccharide types, but significantly caused the difference of monosaccharide proportions in DOPs [32].

As shown in Figure 2c, the absorption peaks at 3424 cm^−1^, 2919 cm^−1^, and 1405 cm^−1^ were attributed to -OH stretching vibration, C-H stretching, flexural vibrations, respectively [33,34], which were the characteristic peaks of polysaccharides. The absorption peak at 1625 cm^−1^ was ascribed to bound water, and the peaks at 1030 cm^−1^ and 1079 cm^−1^ were the characteristics of C-O-H side groups and C-O-C glycosidic rings [35]. In addition, the characteristic peaks at 836 cm^−1^ and 944 cm^−1^ indicated the α- and β-glycosidic bonds in DOPs [36].

### 3.3. 1D ^1^H and 2D COSY Spectra Analysis

**Figure 3:**
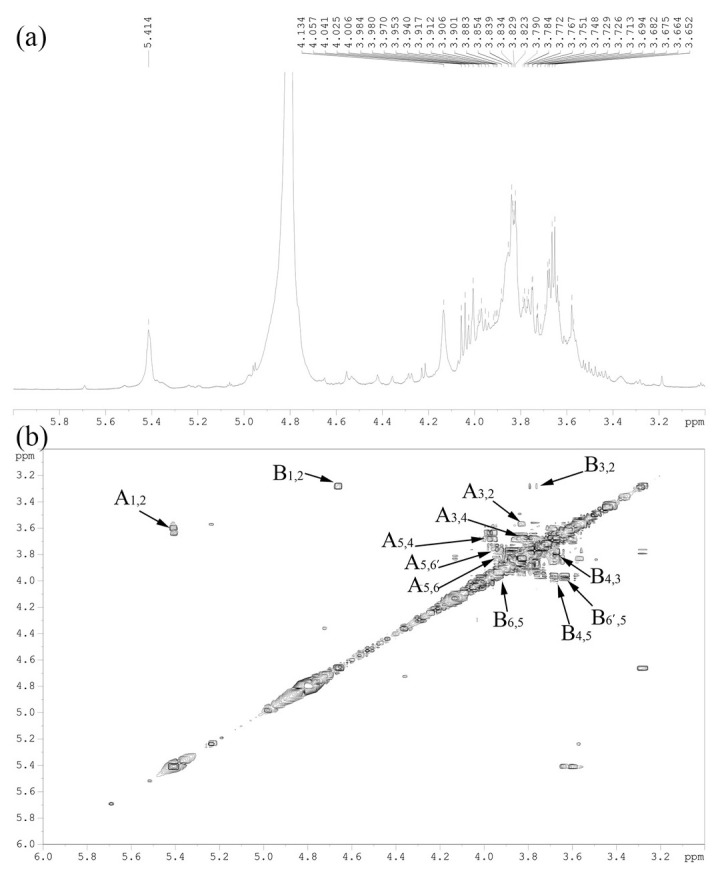
The 1D ^1^H (**a**) and 2D COSY (**b**) spectra of DOPs (solvent: D_2_O).

The 1D ^1^H and 2D COSY spectra of DOPs were shown in Figure 3a,b. As displayed, the main anomeric protons signals at around 5.41 ppm and 4.76 ppm were attributed to α-type Glc glucosidic bonds and β-type Man glucosidic bonds, respectively. Besides, in Figure 3b, “A” represents for the α-type Glc glucosidic bonds, “B” represents for the β-type Man glucosidic bonds, the first number after A/B represents for the x-coordinate, and the second number after A/B represents for the y-ordinate. Therefore, the cross-absorption peaks of adjacent hydrogen atoms of A/B were identified, which would be responsible for the further determination for chemical shifts of carbon atoms in each monomer.

### 3.4. 2D HSQC and HMBC Spectra Analysis

**Figure 4:**
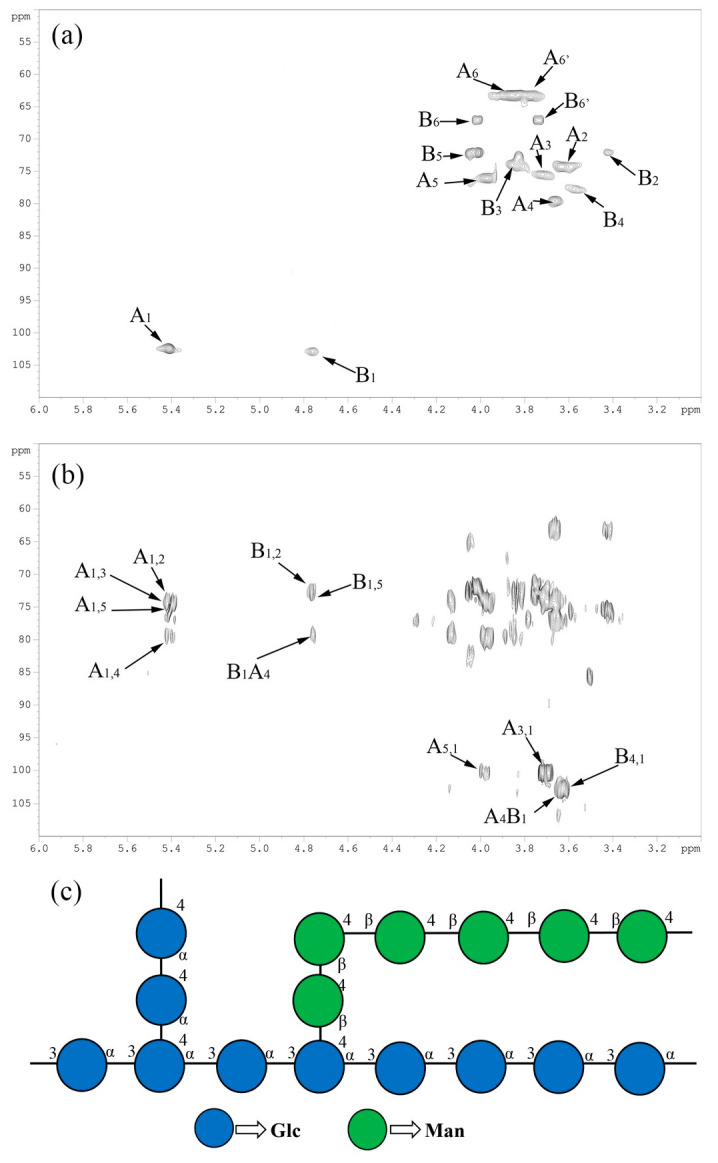
The 2D HSQC (**a**)/HMBC (**b**) spectra and possible chemical structure (**c**) of DOPs.

The 2D HSQC and HMBC spectra of DOPs were displayed in Figure 4a,b, the “A” and ”B” represented α-type Glc glucosidic bonds and β-type Man glucosidic bonds, respectively. As displayed in Figure 4a, the numbers after A/B represent for the carbon and hydrogen serial numbers of monosaccharides, and the chemical shifts of C_1_~C_6_ and H_1_~H_6_ were determined combining with the COSY spectrum analysis, and the results were analyzed and shown in Table 1.

As demonstrated in Figure 4b, the first number after A/B represents for the x-coordinate (^1^H-NMR), and the second number after A/B represents for the y-ordinate (^13^C-NMR), then the hydrocarbon remote cross absorption peaks of A_1,2_, A_1,3_, A_1,5_, A_1,4_, A_5,1_, A_3,1_ were identified, which indicated that the DOPs was composed of α-(1,3)-Glc*p* and α-(1,4)-Glc*p*. While the existence of B_1,2_, B_1,5_, B_1_A_4_, A_4_B_1_, B_4,1_ suggested that the DOPs was comprised of β-(1,4)-Man*p*, which connected to the main chain by C_4_ of Glc [34]. These results demonstrated that DOPs was made up of α-(1,3)-Glc*p* as the main skeleton, and the α-(1,4)-Glc*p* and β-(1,4)-Man*p* as the branches. Finally, the possible chemical structure of DOPs was drawn and displayed in Figure 4c.

### 3.5. Effects of DOPs on Organs Indices

**Figure 5:**
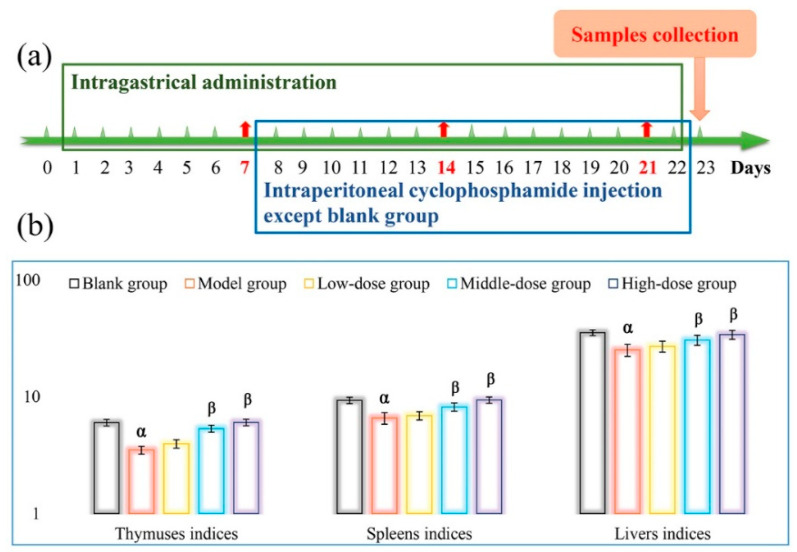
Flow chart of animal experiment (**a**) and effects of DOPs on the organ indices (**b**). Note: ^α^ *p* < 0.05 vs. the blank group; ^β^ *p* < 0.05 vs. the model group.

A decline of apparatus exponential is usually considered as a vital signal for the decrease of immune function in immunosuppressed animals. Thymus and spleen are one of the major immune organs, where immune cells (T and B lymphocytes, macrophages, NK cells, et al.) occur, differentiate and mature, as well as immunity formation [37]. As illustrated in Figure 5, both thymic and splenic indices in the model group were significantly smaller than those in the blank group (*p* < 0.05). Researches indicated that CTX can suppress the differentiation of lymphocytes and reduced their numbers in immune organs, which might be responsible for the decline of spleen and thymus indices [15]. Compared with the model group, both the thymic and splenic indices in the mice treated with DOPs were dose-dependently increased, especially in the medium- and high-dose DOPs groups (*p* < 0.05), indicating that DOPs could effectively relieve CTX-induced damage of immune organs. Additionally, the effects of DOPs oral treatment on liver index in immunosuppressed mice were also evaluated. As indicated, the liver index of mice in the model group were remarkably declined compared with the blank control (*p* < 0.05), verifying severe side effects of CTX on mice organs. In contrast, various doses of DOPs significantly reduced the toxicity of CTX and enhanced the liver indices of mice.

### 3.6. Immune Cells Activities

**Figure 6:**
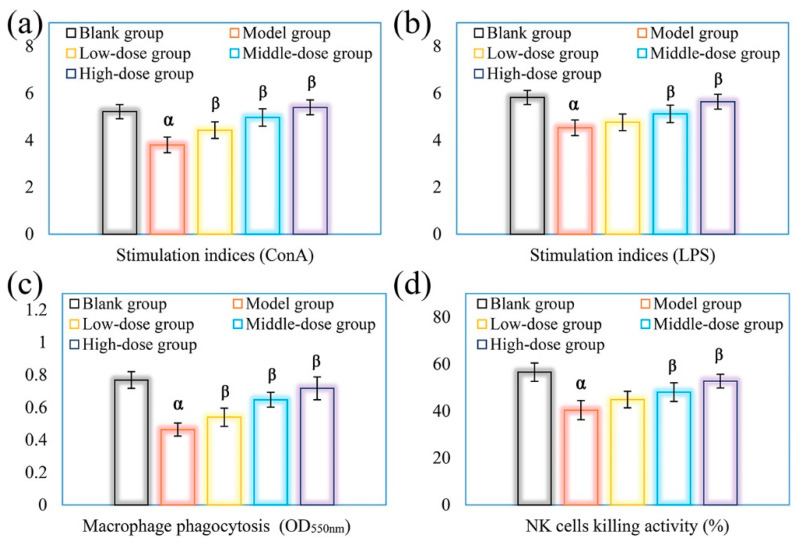
Effects of DOPs on the splenic T lymphocytes proliferations induced by Con A (**a**), the splenic B lymphocytes proliferations induced by LPS (**b**), phagocytic activity of peritoneal macrophages (**c**), and splenic NK cytotoxic activity (**d**) in immunosuppressed mice. Note: ^α^ *p* < 0.05 vs. the blank group; ^β^ *p* < 0.05 vs. the model group.

The spontaneous lymphatic proliferation is usually applied to evaluate cellular immunologic response. Generally, T and B lymphocytes are nonspecific in immune system, which of proliferation can be stimulated by Con A and LPS, respectively [38]. NK cells are regarded as a kind of cytotoxic lymphocytes, which have a crucial part in resisting the attraction of viruses and tumors in organism [39]. Macrophages participate in non-special immunity, and their phagocytosis exert an important role in inducing immune response and maintaining the homeostasis of immune system [40]. In the present study, the influences of DOPs on the proliferative activity of splenic T and B lymphocytes, the cytotoxic effect of splenic NK cells, and the phagocytosis of peritoneal macrophages from CTX-immunosuppressed mice were investigated for the evaluation of the immune cells activities. As shown in Figure 6, the activities of three immune cells were all obviously reduced in the model group compared with the blank group (*p* < 0.05), which further verified the immunosuppressive action of CTX on the mice. The significant enhancements of these immune cells activities were observed in immunosuppressed mice treated orally with DOPs with a dose-dependent manner, which exhibited a significant difference, especially in the medium- and high-dose DOPs groups, compared with those in the model group (*p* < 0.05). These results suggested that DOPs could effectively repair cellular immune function in CTX-exposed mice.

### 3.7. Leukocytes Amounts and Cytokines Levels in Peripheral Blood

Cytokines are small soluble proteins which were produced and secreted by various activated non- and immune cells, which can participate in cellular interactions, regulate cell growth, differentiation and maturation to mediate body’s innate and adaptive immunity [41]. As we all known, cytokines such as interleukin-2 (IL-2), interferon-γ (IFN-γ), and tumor necrosis factor alpha-α (TNF-α) are mainly produced by Th1 cells, which involve in cellular immune response of body [42]. Therefore, the serum cytokines (TNF-α, IFN-γ and IL-2) expressions were detected by ELISA.

As indicated in Table 2, the levels of three serum cytokines remarkably decreased in model group compared with blank group (*p* < 0.05), confirming the significant suppressive effects of CTX on the secretions of these cytokines in sera. In contrast, DOPs treatments exhibited the dose-dependent enhancement of sera’s TNF-α, IFN-γ and IL-2 levels, which were observably higher than that of model group (*p* < 0.05), which indicated that DOPs can alleviate the decline of sera’s cytokines in immunosuppressed mice.

### 3.8. Antioxidant Activities In Vivo

The production of excess free radicals is closely correlated with various diseases, which can destroy the body’s immune cells. Therefore, the antioxidant defenses are extremely important for normal immune system functions. Natural polysaccharides were proved to have oxidation resistance by enhancing the activities of the antioxidant enzymes such as SOD and GSH-Px [43]. SOD is considered as a classical scavenger of free radicals under oxidative stress and exists widely in body [19]. GSH-Px is an important peroxidase that can reduce hydrogen peroxide to H_2_O in cytosol to protect the immune cells against superoxide [44]. MDA, as the final product of lipid peroxidation, is usually served as an typical biomarker of lipid peroxidation [45]. In the present study, the effects of DOPs on SOD and GSH-Px activities and MDA contents in sera and livers of CTX-induced immunosuppressed mice were displayed in Table 3 and Table 4.

The levels of SOD and GSH-Px in serum and livers of model group were lower (*p* < 0.05), whereas the MDA contents were higher than the blank group (*p* < 0.05), indicating the inhibitory effects of CTX on the antioxidant functions of immunosuppressed mice. Interestingly, DOPs treatment lead to a significant improvement of SOD and GSH-Px levels (*p* < 0.05), and an observable decline of MDA contents (*p* < 0.05) in sera and livers with a dose-dependent manner compared to model group. These results confirmed that DOPs were capable of effectively protect immunosuppressed mice from oxidative stress, which might be attributed to maintain the redox balance and improve the immune system function in the body.

## 4. Conclusions

In the present study, a novel low-molecular weight polysaccharide (DOPs) was isolated from *D. officinale*. The results demonstrated that DOPs mainly consisted of glucose and mannose at a molar ratio of 1.00:5.78 with the average molecular weight of 4.56 × 10^3^ Da, which was composed of α-(1,3)-Glc*p* as the main skeleton, and α-(1,4)-Glc*p* and β-(1,4)-Man*p* as the branches. Animal experiment results showed that DOPs exhibited significant immunomodulatory activity on CTX-induced immunosuppressive mice via improving the immune organs indices, increasing the immune cells abilities, promoting the serous cytokines productions. Additionally, DOPs could also present effective enhancement on antioxidant capacities of immunosuppressed mice. These results could provide novel insights into comprehensive utilization of *D. officinale*.

## Figures and Tables

**Table 1:** Chemical shifts of C_1_~C_6_ and H_1_~H_6_ in monomers of DOPs.

Monomers	H_1_/C_1_ ppm	H_2_/C_2_ ppm	H_3_/C_3_ ppm	H_4_/C_4_ ppm	H_5_/C_5_ ppm	H_6,6′_/C_6_ ppm
α-type Glc linkages	5.41 102.63	3.61 74.42	3.72 75.54	3.66 79.72	3.97 76.23	3.86, 3.77 63.53
β-type Man linkages	4.77 103.11	3.43 72.19	3.83 74.14	3.57 77.95	4.02 72.26	4.02, 3.74 67.23

**Table 2:** Cytokines expressions levels of mice in each group.

Groups	TNF-α (ng/L)	IFN-γ (ng/L)	IL-2 (ng/L)
Blank group	269.44 ± 13.81	303.67 ± 20.72	168.94 ± 9.68
Model group	218.25 ± 14.05 ^α^	236.75 ± 14.84 ^α^	136.49 ± 9.83 ^α^
DOPs group (50 mg/kg)	232.31 ± 17.12 ^β^	259.11 ± 15.37 ^β^	152.80 ± 8.76 ^β^
DOPs group (100 mg/kg)	253.37 ± 12.75 ^β^	262.75 ± 13.77 ^β^	161.08 ± 11.13 ^β^
DOPs group (150 mg/kg)	265.83 ± 14.01 ^β^	288.42 ± 19.37 ^β^	170.29 ± 13.82 ^β^

Note: ^α^, *p* < 0.05 compared with the blank group; ^β^, *p* < 0.05 compared with the model group.

**Table 3:** Antioxidant enzymes activities and MDA contents of mice sera.

Groups	SOD (unit/mL)	GSH-Px (unit/mL)	MDA (nmol/mL)
Blank group	235.92 ± 11.04	421.73 ± 21.36	3.98 ± 0.25
Model group	186.53 ± 10.16 ^α^	351.16 ± 17.39 ^α^	5.99 ± 0.36 ^α^
DOPs group (50 mg/kg)	203.41 ± 11.23 ^β^	375.98 ± 16.48 ^β^	5.53 ± 0.47 ^β^
DOPs group (100 mg/kg)	216.33 ± 9.88 ^β^	402.42 ± 18.49 ^β^	4.83 ± 0.34 ^β^
DOPs group (150 mg/kg)	236.52 ± 12.49 ^β^	427.58 ± 20.82 ^β^	4.07 ± 0.28 ^β^

Note: ^α^, *p* < 0.05 compared with the blank group; ^β^, *p* < 0.05 compared with the model group.

**Table 4:** Antioxidant enzymes activities and MDA contents of mice livers.

Groups	SOD (Unit/mL)	GSH-Px (Unit/mL)	MDA (nmol/mL)
Blank group	363.39 ± 17.69	836.75 ± 38.31	8.87 ± 0.42
Model group	273.51 ± 16.98 ^α^	762.31 ± 37.87 ^α^	13.95 ± 0.78 ^α^
DOPs group (50 mg/kg)	296.53 ± 16.29 ^β^	795.34 ± 36.92 ^β^	11.56 ± 0.69 ^β^
DOPs group (100 mg/kg)	326.86 ± 14.96 ^β^	816.03 ± 39.11 ^β^	9.93 ± 0.51 ^β^
DOPs group (150 mg/kg)	359.45 ± 15.48 ^β^	837.51 ± 42.67 ^β^	8.56 ± 0.40 ^β^

Note: ^α^, *p* < 0.05 compared with the blank group; ^β^, *p* < 0.05 compared with the model group.

## Data Availability

Data sharing not applicable. No new data were created or analyzed in this study. Data sharing is not applicable to this article.
